# Online housing search: A gravity model approach

**DOI:** 10.1371/journal.pone.0247712

**Published:** 2021-03-24

**Authors:** Joep Steegmans, Jonathan de Bruin

**Affiliations:** 1 Utrecht School of Economics, Utrecht University, Utrecht, The Netherlands; 2 Research and Data Management Services, Utrecht University, Utrecht, The Netherlands; Universitat de Valencia, SPAIN

## Abstract

In this paper we apply a gravity framework to user-generated data of a large online housing market platform. We show that gravity describes the patterns of inflow and outflow of hits (mouse clicks, etc.) from one municipality to another, where the municipality of the user defines the origin and the municipality of the property that is viewed defines the destination. By distinguishing serious searchers from recreational searchers we demonstrate that the gravity framework describes geographic search patterns of both types of users. The results indicate that recreational search is centered more around the user’s location than serious search. However, this finding is driven entirely by differences in border effects as there is no difference in the distance effect. By demonstrating that geographic search patterns of both serious and recreational searchers are explained by their physical locations, we present clear evidence that physical location is an important determinant of economic behavior in the virtual realm too.

## 1 Introduction

Online housing platforms have come to play an important role in housing search. It has led to novel user-generated data, which can be used to analyze online search strategies. The data allow for testing the extent to which mainstream economic theories are fit for the new digitalized context [1]. In line with this, we apply a gravity model to online housing search behavior. Of particular interest is the distinction between serious and recreational searchers as not every platform user has the intention of buying a house. The comparison of serious and recreational searchers aids in identifying the determinants of online search flows.

Gravity models have been successfully applied in the international trade literature ever since the seminal contributions of Tinbergen [2]. The standard model explains bilateral trade flows between countries in terms of their economic masses and the distance between them. The emergence and development of the Internet has led to a new field of research where the reduction in search costs related to online markets are studied. It is hypothesized that Internet usage reduces transaction costs and diminishes the effects of distance. Recent studies do not show evidence of the ‘death of distance’ [3, 4]. However, for goods bought online the effect of distance is substantially smaller than for goods bought offline [4].

Our research builds upon these studies: we use a gravity framework to explain inflows and outflows of hits (mouse clicks, page views, etc.) on the largest housing platform in the Netherlands, Funda.nl. The user’s municipality functions as the origin or ‘export’ location and the municipality of the property that is viewed functions as the destination or ‘import’ location. We use the platform’s user-generated data to study search behavior. In particular, we investigate the extent to which distance and border effects explain existing search flows in this type of big data. The sheer size of the data facilitates estimation as zero flows from one location to another are rare. The rich data also allow us to differentiate between serious searchers and recreational searchers. Serious searchers are the platform users that have the intention of buying a house; they are the potential house buyers [5]. Recreational searchers, on the contrary, use the platform as a pleasant pastime without plans of buying a house.

A major contribution with respect to the use of gravity models in the digital age has been made by Blum and Goldfarb [6]. They study the consumption of digital goods over the Internet. The application of a gravity equation to digital goods is interesting because the digital goods have no trading costs. Blum and Goldfarb demonstrate that distance still matters. They find that, all else equal, American Internet users are more likely to visit websites from nearby countries. Blum and Goldfarb show that the law of gravity holds for “taste-dependent” goods, among which, music, games, and pornography, while it does not hold for “non-taste-dependent” goods, such as software. Still, in a world with exponential technological change the study of Blum and Goldfarb [6] seems from a different era already: all the Internet users in the 1999-2000 sample were dial-up users and Google had a market share for search engines of only 0.6 percent.

Hortaçsu et al. [3] study the geography of trade in online transactions, based on data from eBay and MercadoLibre. They find that “distance continues to be an important deterrent to trade between geographically separated buyers and sellers, though to a lesser extent than has been observed in studies of non-Internet commerce” (p. 53). Lendle et al. [4] also use eBay data to study the effect of distance on international trade flows. They, too, find that distance matters even though the distance effect of online trade is 65 percent smaller than that of offline trade. They argue that reduced search costs limit the distance effects.

Lendle et al. [4] argue that the Internet indeed facilitates a reduction in search costs and that online marketplaces, such as eBay, can be considered frictionless in this respect. They argue that ‘information frictions’ remain and that these drive the distance effect of eBay trades. Apart from that, taste differences can also explain part of the distance effect. Following work of Rauch [7], Dasgupta and Mondria [8] present a model that uses information frictions, the costs of processing information, to explain traditional trade flows between countries.

Our research demonstrates that the gravity framework can successfully be applied to housing search of both serious and recreational searchers. We show its usefulness at a particularly low level of aggregation (i.e., the municipality level). Even the simplest of gravity models, with only mass, distance, and border variables, explains close to 80 percent of the bilateral flows—even when no distinction is made between serious and recreational searchers. Including origin and destination fixed effects increases the explanatory power of the model even further. All findings are robust to the estimation procedure: least squares estimation with fixed effects and Poisson pseudo maximum likelihood lead to similar findings.

The paper is organized as follows. In Section 2, we define the gravity framework that originates from the international trade literature. In Section 3, the data set and the variables are discussed. Section 4 provides the empirical model that we will estimate and discusses the estimation techniques that can be used. In Section 5, we present the results and their robustness by examining the effects of measurement error in the user’s location. Section 6 summarizes our findings and concludes.

## 2 Theoretical background

We use the gravity model from the international trade literature as our starting point as this has also been used for Internet trade and digital goods. The gravity model is regularly claimed to be one of the empirically most successful models in economics [9]. While gravity models have been applied to bilateral trade since Tinbergen [2] the theoretical (microeconomic) foundations only followed decades later. The first theoretically grounded gravity model, a so-called structural gravity model, was provided by Anderson [10]. Still, structural gravity models only became popular after the seminal contributions of Eaton and Kortum [11] and Anderson and Van Wincoop [9]. It is the latter study that seems to have become the standard in the literature. It is for that reason that we will use the Anderson-Van Wincoop model as our point of departure.

Anderson and Van Wincoop [9] make the convincing argument that bilateral trade flows should be studied relative to the average trade barriers with all other regions. After all, higher average trade barriers with all other regions will increase trade flows between a given bilateral pair. Anderson and Van Wincoop [9] refer to these average trade barriers as “multilateral resistances”. Applying a structural gravity model implies that the model gets a general equilibrium interpretation; that is, it recognizes that a change in trade costs between region *i* and *k* affects the trade flow between *i* and *j*. Hence estimation leads to coefficients that allow for comparative statistics regarding, for instance, border effects.

Anderson and Van Wincoop [9] derive the following structural gravity model:

$$\begin{array}{c} x_{ij}=\frac{y_{i}y_{j}}{y^{W}}{\left(\frac{t_{ij}}{\Pi_{i}P_{j}}\right)}^{1-\sigma } \end{array}$$ (1)

where *x*_*ij*_ is exports from region *i* to region *j*, *y*_*i*_, *y*_*j*_, and *y*^*W*^ are nominal income in, respectively, regions *i*, *j*, and the world, *t*_*ij*_ are trade costs between region *i* and *j*, *σ* is the elasticity of substitution for import goods, and *Π*_*i*_ and *P*_*j*_ are multilateral resistance terms of the exporting region *i* and the importing region *j*. Even though Anderson and Van Wincoop [9] use *P*_*i*_ for the outward multilateral resistance, it has become the standard to use *Π*_*i*_ instead.

The determinants of trade costs, *t*_*ij*_, include common factors between the trading partners. They include both geographical and cultural factors. These determinants include bilateral distances and contiguity as well as, for instance, common language, colonial history, and shared cultural identities [12]. Broadly defined trade costs also include information costs; these costs, however, are generally hard to measure [13]. Trade costs are modelled log-linearly leading to a specification of the form:

$$\begin{array}{c} t_{ij}=d_{ij}^{\rho_{1}}exp(\rho_{k}z_{ij}^{k}) \end{array}$$ (2)

where *d*_*ij*_ is the bilateral distance between region *i* and *j*, $z_{ij}^{k}$ is a set of dummies capturing common traits (with *k* = 2, …, *K* indicating the trait). In the case of Anderson and Van Wincoop [9]*z*_*ij*_ contains bilateral distances and border barriers.

Following Anderson and Van Wincoop [9], we impose unitary coefficients for the economic mass variables. Log-linearizing Eq (1) then leads to:

$$\begin{array}{c} ln\left(x_{ij}\right)=k+ln\left(y_{i}\right)+ln\left(y_{j}\right)+\left(1-\sigma \right)ln\left(t_{ij}\right)-\left(1-\sigma \right)ln\left(\Pi_{i}\right)-\left(1-\sigma \right)ln\left(P_{j}\right) \end{array}$$ (3)

where *k* = −*ln*(*y*^*W*^) is a constant. Eq (3) illustrates why Head and Mayer [12] talk about the “multilateral resistance/fixed effects revolution” (p. 135) when referring to the contributions of Anderson and Van Wincoop [9] and Eaton and Kortum [11]. By providing clear theoretical foundations to the gravity model, the seminal papers demonstrated that importer and exporter fixed effects are an essential part of gravity model estimation, thereby altering the estimation methods used in empirical studies.

While the Anderson and Van Wincoop model is generally used to study international trade (between countries), it can also be used to study intranational trade (within a country) [9]. Gravity “appears to work well at almost any scale” [14]. Lower-scale gravity model applications include, for instance, analyses of provincial border effects in Canada [15] and state border effects in the United States [16]. Trade flows of Canada’s provinces have also been used to model intra-regional, inter-regional and international frictions simultaneously [17]. The analysis of interstate migration in Mexico is a non-trade (intranational) illustration [18].

Following the theoretic foundations of gravity models in bilateral trade, similar attempts have been made for other applications of the gravity model. To name a few, Anderson [14] provides micro-foundations for migration and foreign direct investment, Okawa and Van Wincoop [19] provide theoretical foundations for bilateral financial holdings, Egger et al. [20] provide them for services, and Persyn and Torfs [21] provide a micro-foundation for commuting. From an empirical point of view, it should be noted that, although the micro-foundations differ, the derived gravity model is the same. Allen et al. attempt to incorporate a wide class of general equilibrium models, combining demand and supply functions, into a ‘universal gravity’ theory [22]. Still, differentiating between the underlying conditions of “observationally equivalent” gravity models [14] is beyond the scope of this article.

As mentioned before, we build on the methodological contribution made by Anderson and Van Wincoop [9]. We will discuss the consequences regarding the interpretation of our model specification in Section 4, but before we do so we will demonstrate that the descriptives of the data fit the gravity model particularly well.

## 3 Data

### 3.1 The Funda platform

The data that we use for the analyses originate from Funda.nl, the largest housing platform in the Netherlands. Funda was established by the Dutch Association of Realtors (NVM) in 2001 as an online platform for properties for sale. During the early years Funda listed only the properties of NVM realtors, covering roughly seventy percent of the Dutch property market. Competing realtor associations gained access to the platform in 2009. Funda focuses on the owner-occupied market although the number of listed rental properties is on the rise. During the years Funda has become the undisputed, dominant housing website in the Netherlands [23]. The data set is predominantly user-generated: it contains detailed analytics of the website users’ behavior. The non-user-related data consist of the listings on the Funda website (i.e., houses and apartments).

The data set that we use covers the six month period from January 1, 2018 to June 30, 2018. The data are collected by Google Analytics, Google’s web analytics service. Funda uses these data for statistics to gain insights in the platform’s usage, to maintain platform stability, and for product development [24]. The Google Analytics data contain dimensions and metrics. Dimensions are attributes of the data, such as the location a session originates from and the user’s device. Metrics are quantitative measurements, such as the number of hits that are made and the time spent on the website [25]. Apart from the standard dimensions and metrics, there are also custom dimensions. In the case of Funda, these include information on the object that is viewed, such as the location of the object, and whether the object is for sale or to be rented.

A website interaction that results in data being sent to Google Analytics is called a hit. Hits can be divided into events and page views. Events include, for instance, link clicks, form submissions, and video plays while page views indicate that a new webpage is loaded [25]. Hits from the same user that take place within a given time span are grouped together in a session; a session is closed after thirty minutes of inactivity. The data set is a combination of dimensions and metrics at the hit level, the session level, the user level, and the object level. The underlying data set that we use consists of 1.097 billion hits related to 279,941 houses and apartments that have been listed.

One of the main user dimensions in our analyses is the geolocation, the approximate location from where the individual searches, which is derived from the IP address [26]. The client (i.e., the user’s browser) sends an information request to the server to send a copy of the part of the website that is requested. In this request information is included on, for instance, the hostname, browser, language settings, and IP address; information that is collected by Google Analytics. The included IP address is provided with a location by Google Analytics. It seems that Google Analytics uses a passive, database-driven IP geolocalization method—in contrast to an active IP geolocation method, using network delay measurements (for more technical details on these methods, see: [27, 28]).

The IP-based locations are approximations. Particularly at the lowest level of the geographical indicator (i.e., villages, towns, and cities) these errors may be substantial. Google Analytics refers to this geographical indicator as the “city”. However, in the Netherlands this regional indicator includes villages such as Biervliet, Retranchement, and Sint Kruis, which, on January 1, 2018, had 200, 313, and 329 inhabitants, respectively [29]. There is another reason why we will treat the geolocations cautiously: the users’ IP addresses have been made compliant with the General Data Protection Regulation (GDPR) by replacing the last bits (in binary form) from the IP address with zeros before geolocations are added. For instance, the IP address 131.211.209.183 (in decimal form) would be changed into 131.211.209.000. The ‘anonymization’ of IP addresses is part of a wide series of measures set in motion by the GDPR. Furthermore, the (anonymized) IP addresses are not included in the data; only the corresponding geolocation is included. For details on how Funda deals with the GDPR, see [24].

This leads to three possible user (geo)locations. The first is the unobserved (true) physical location, the second is the geolocation based on the full IP address, and the third is the geolocation based on the anonymized IP address. While locational errors in our data are partly mitigated by aggregating villages and towns into municipalities, we take advantage of the fact that location accuracy is device and network domain specific; that is, the error between the physical location and the full IP address is depending on the device used (particularly, mobile vs. non-mobile), while differences between full and anonymized IP addresses are depending on, for instance, network domains. We discuss the matter in detail in the Appendix: IP anonymization.

### 3.2 Hit flows

Our focus is on hit flows from the searcher’s location to the location of the object that is viewed. In other words, we trace both the origin of a hit and the destination of it. The hits are aggregated at the municipality level to provide us with a data set containing the inflow and outflow of hits, from and to municipalities in the Netherlands. We have additional information on both the user and the object. We observe, for instance, the device of the platform user and the time spent online. Regarding the object, we know whether it is an apartment or a house, whether it is for sale or for rent, and whether it is still on the market.

The hit flows are defined based on the origins and the destinations of the hits. Consequently, only hits of which both the origin and the destination are observed are included in our analyses. In practice this means that only hits on object pages are included: start pages, query result lists, etc. cannot be contributed to one particular object location. Furthermore, we only include hits where both the origin and the destination is Dutch. We condition on objects still being on the market as sold properties are less interesting from a search perspective. Besides, listings of sold properties have not been constant over time. For instance, starting 11 April, 2017, Funda started to keep sold properties longer accessible for platform users.

We have data of in total 382 origin municipalities corresponding to 1,181 distinct Google locations in the Netherlands. We have no data for six small (origin) municipalities; Google Analytics does not include villages within the municipality borders of Ameland, Schiermonnikoog, Vlieland, Rozendaal, Haarlemmerliede en Spaarnwoude, and De Marne. It should be noted that the flows from these municipalities are missing; it does not imply that the flows are zero. For the destination municipalities, we observe houses and apartments in all 388 Dutch municipalities. The total number of flows is, therefore, 148,216 (382*388), including 382 internal flows.

**Fig 1:**
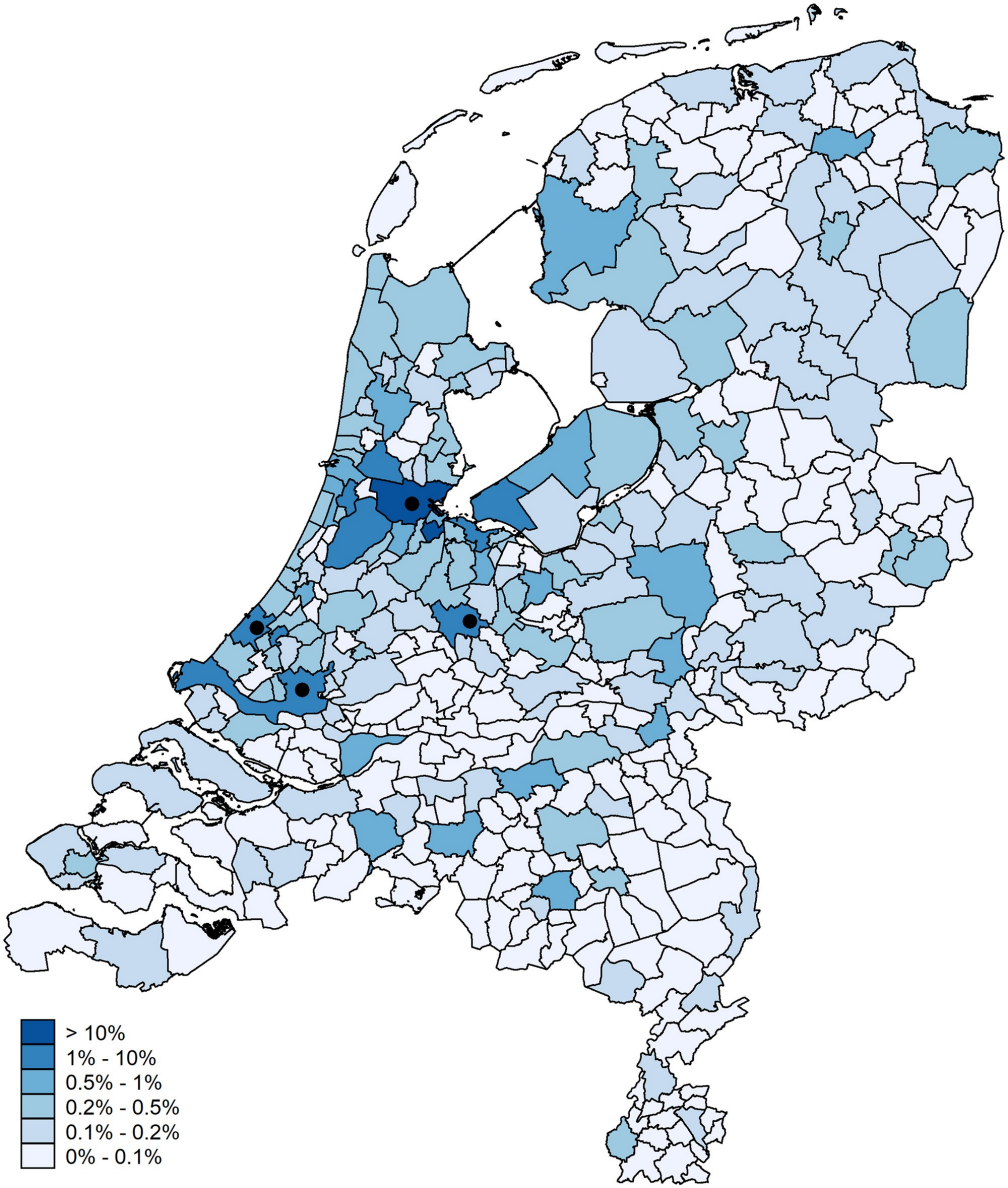
Hits from Amsterdam. Digital geometry (i.e., the shapefile) obtained from CBS/Kadaster [30]).

**Fig 2:**
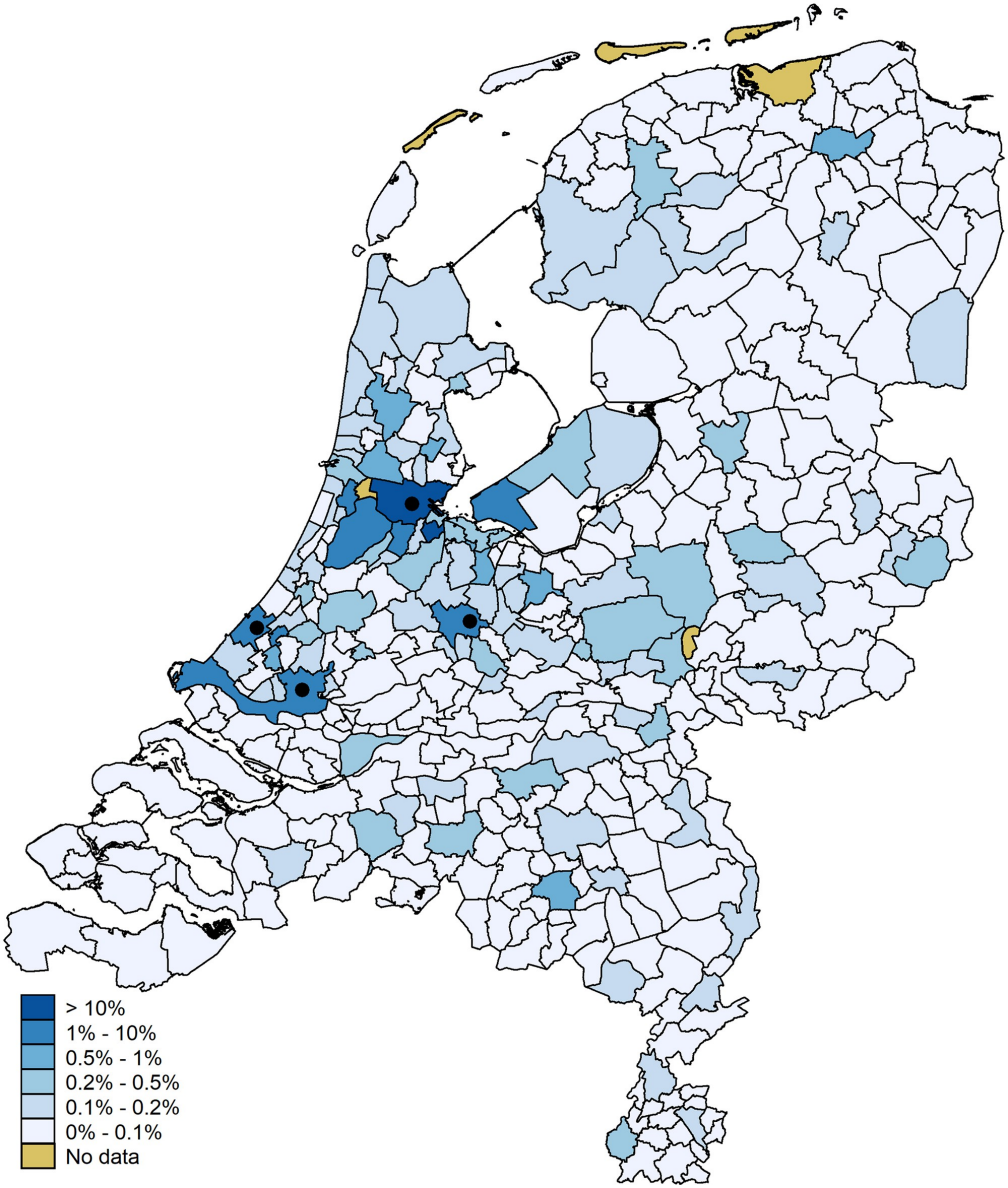
Hits to Amsterdam. Digital geometry (i.e., the shapefile) obtained from CBS/Kadaster [30]).

Fig 1 illustrates the flow data by presenting the relative outflow of hits from the municipality of Amsterdam (the origin) to the other municipalities (the destinations), where the four marker points show the location of the four largest cities in the Netherlands. The City of Amsterdam is indicated by the most North of the marker points. The other three markers are, from West to East, the cities of The Hague, Rotterdam, and Utrecht. The map shows that, expressed as a percentage of the total flow from Amsterdam, flows are larger to both nearby municipalities and to larger municipalities. The latter is the case for, for instance, The Hague, Rotterdam, and Utrecht. Similarly, Fig 2 presents the flows of the other municipalities to Amsterdam. The map shows, again, that nearby and larger municipalities contribute substantially more to the total flow that has Amsterdam as the destination. Both figures thus illustrate the mass and distance effects inherent to gravity models: municipalities closer to Amsterdam have larger outflows (and inflows) while the same holds for larger municipalities.

### 3.3 Mass and distance

For illustrative purposes, we define the mass of the origin as the number of (unique) users in the origin municipality. After all, the total (outward) flow from a municipality is closely related to the corresponding number of platform users. In contrast, we define the mass of the destination as the number of listed objects in the destination municipality. The (inward) flows towards municipalities are, arguably, closer related to the number of houses and apartments in the destination municipality than they are to the number of platform users residing in the destination municipality. (Alternative mass variables—based on the number of inhabitants, the number of households, or even the aggregate number of hits—do lead to similar figures and do not affect the estimation results.) From an empirical point of view, it is worth realizing that in estimation the mass effects are going to be captured by origin and destination fixed effects.

Distance is defined as the Euclidean distance between the centroids of the municipalities. The curvature of the earth within the Netherlands is negligible as the country is small. The centroids are coordinates expressed in the Dutch coordinate reference system (Rijksdriehoeksmeting; RD). We approximate the internal distances by what has become the standard in the gravity literature: 23A/π where *A* is the surface area. The approximation was introduced by Head and Mayer [31]. The approximation relies on the surface areas being known. In our study, it will turn out that the findings are unaffected by including or excluding the internal flows. This is largely due to the small number of internal flows compared to the total number of flows (382 out of 148,216). Thus, although in terms of hits their share is substantially larger, the role of internal flows is limited from an empirical point of view.

**Fig 3:**
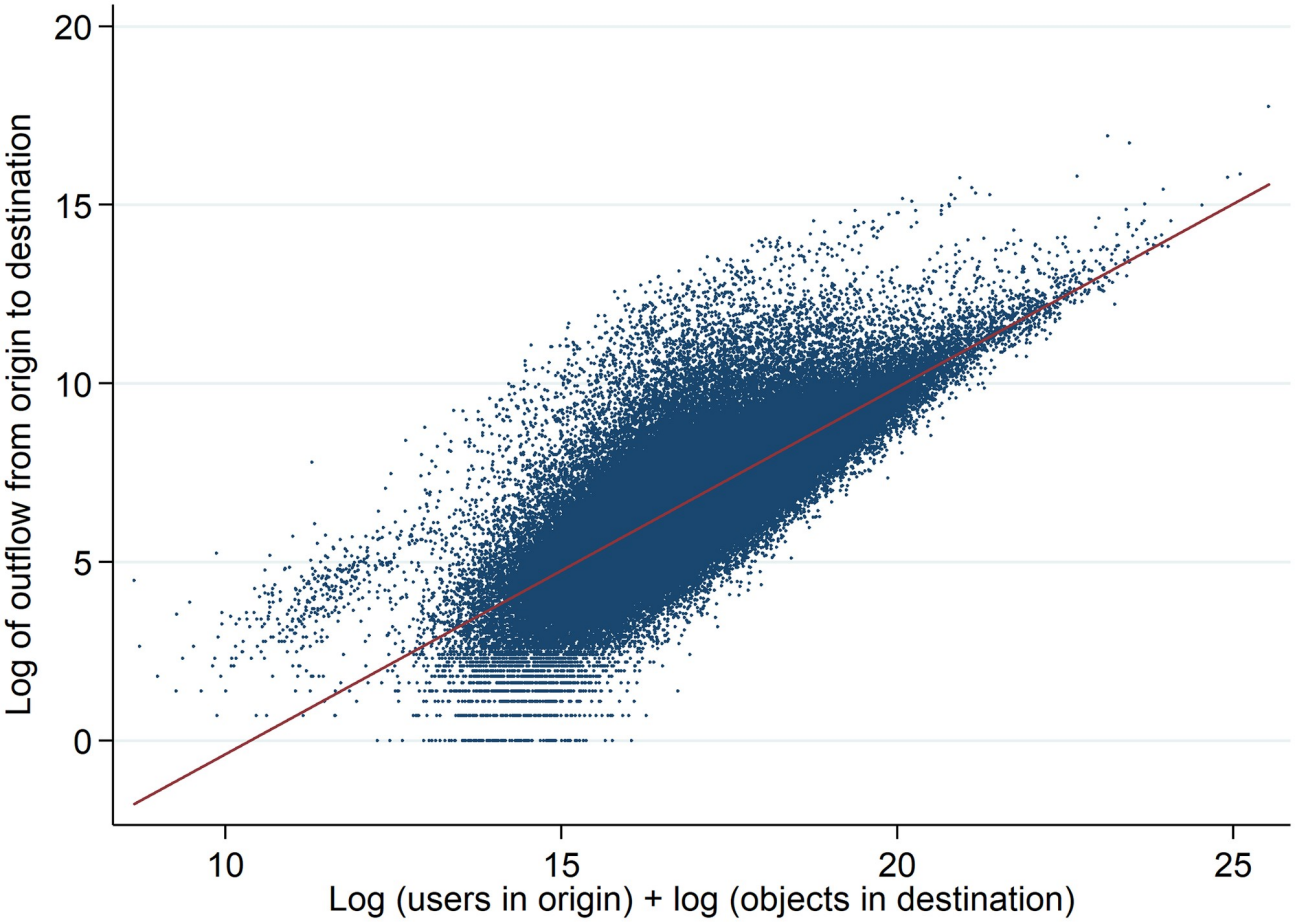
Hit flows between municipalities in relation to mass.

**Fig 4:**
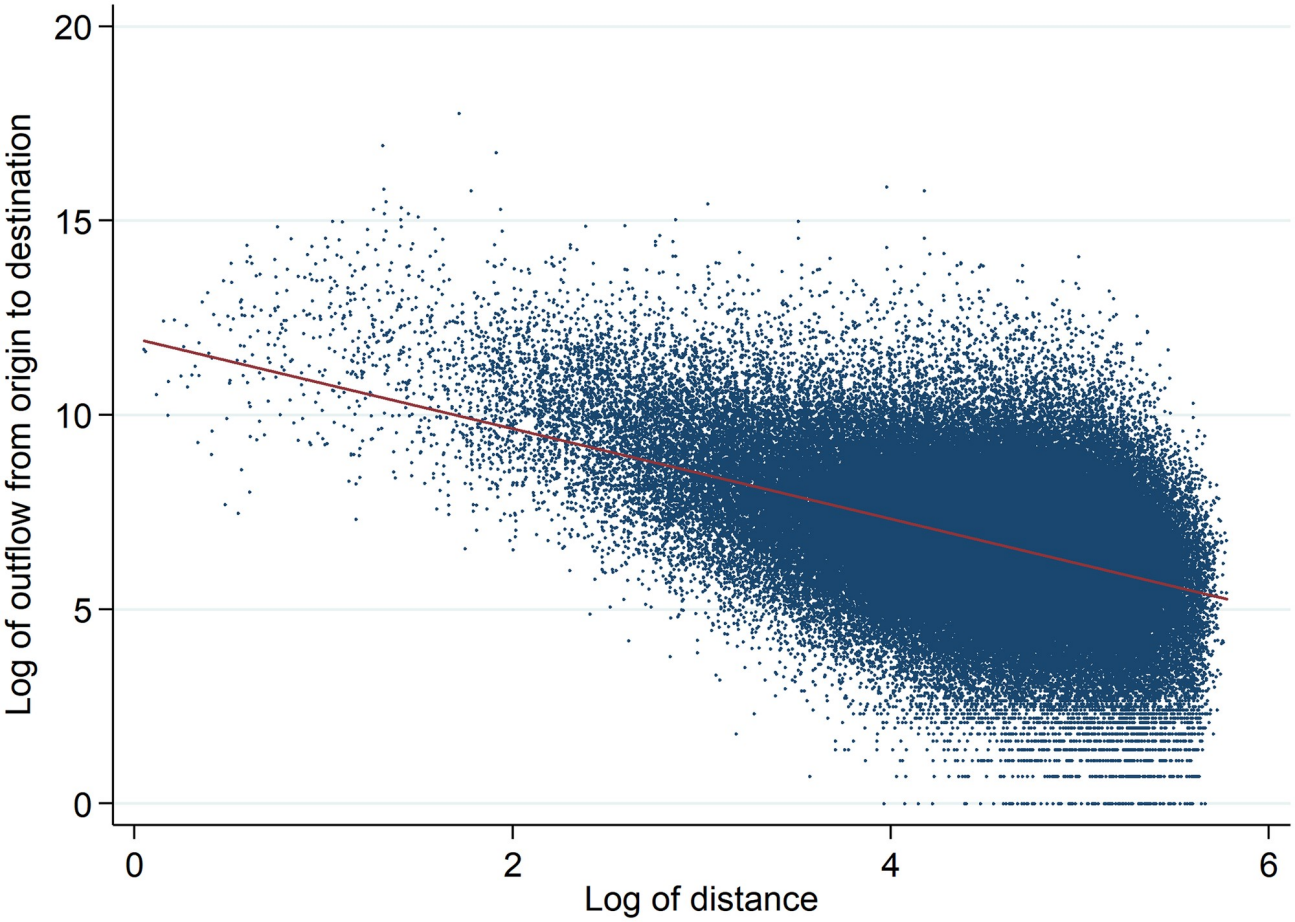
Hit flows between municipalities in relation to distance.

Fig 3 shows the relation between the size of the hit flow from one municipality to another and the product of the number of users in the origin and the number of listings in the destination. The figure clearly demonstrates the positive correlation between the size of the hit flow and the masses of the origin and destination municipalities, illustrating the gravity mass effect. Fig 4 shows the relation between the volume of the flows and the distances between the municipalities, illustrating the gravity distance effect. The figure clearly illustrates decreasing flows for greater distances.

### 3.4 Serious versus recreational search

The intentions for using the platform differ widely among individuals. The intentions stretch the full spectrum from serious to recreational. As noted by Rae [5], recreational or curiosity searchers should be distinguished from potential house buyers. We will distinguish different types of users based on actions they take on the online platform. The corresponding subsamples allow us to distinguish the search behavior of the serious searchers from the recreational searchers. We do not expect that the actions perfectly identify all of the users’ intentions at the individual level. However, we do expect that at the aggregate level the actions and the intentions will show substantial correlations. We will estimate separate gravity equations for the groups that we observe.

Information on the actions that signal the searcher’s intention is provided in Table 1. Column 1 provides the numbers for the full data set, which includes both recreational searchers and serious searchers. The columns 2 to 8 of Table 1 show the shares for different subgroups. From left to right we expect the user intentions to become more serious. For instance, to make use of the basic Funda services, such as making a list of favorite houses and saving preferences, users need to register and create an account. Hence we consider the creation of an account the first, and lowest, barrier. Using the mortgage tool to calculate one’s predicted monthly mortgage costs is expected to correlate with more serious buying intentions although it may still be curiosity driven. Similarly, clicking on ‘Show telephone number’ in order to be able to call the real estate agent seems to be an indication of increasing interest while still being low barrier as users may not actually call. Table 1 shows that these first three subsamples consist of about twenty percent of all hits. Similar percentages are found when looking at events (the aforementioned subgroup of hits) or when expressed in terms of the time spent on the website.

**Table 1:** Serious searchers (potential house buyers).

	(1)	(2)	(3)	(4)	(5)	(6)	(7)	(8)
	All	Account	Mortgage	Telephone	Email service	Message	Viewing	Buyer
Hits (×10^6^)	1096.6	244.3	213.8	230.4	118.3	88.2	92.2	4.4
Events (×10^6^)	632.8	150.0	125.7	143.1	70.4	54.3	56.7	2.8
Time on site (×10^6^ hours)	12.6	2.8	2.6	2.9	1.3	1.0	1.1	0.1
Non-zero flows	147,903	143,997	142,317	142,072	135,050	126,757	122,913	43,704
Hits (percent)	100	22.3	19.5	21.0	10.8	8.0	8.4	0.4
Events (percent)	100	23.7	19.9	22.6	11.1	8.6	9.0	0.4
Time on site (percent)	100	22.1	20.2	22.8	10.6	8.0	8.6	0.4
Non-zero flows (percent)	99.8	97.2	96.0	95.9	91.1	85.5	82.9	29.5

*Notes*: Account, users that have registered and created a user account; Mortgage, users that have done the online mortgage calculation; Telephone, users that have pressed the button for the real estate agent’s telephone number; Email service, users that have signed up for an email service of new listings within their preferences; Message, users that have contacted the real estate agent through an online form; Viewing, users that scheduled a viewing with the online tool; Buyer, users that registered themselves as the buyer of a property.

Users that sign up to receive emails containing new listings that fall within specified boundaries are expected to be even more serious; not only do they put in effort once, the emails suggest future effort too. The same holds for users that use a form to send a message to a real estate agent and those scheduling a viewing through the available online tool. These latter three groups make up between eight and eleven percent of all hits. Again, similar percentages are found in terms of events and time on site. The most serious searchers that we observe are the users that eventually register themselves as the buyer of a property. The additional effort they have to put in suggests that indeed they bought the property. As indicated by Table 1, the hits made by users from this latter group make up only 0.4 percent of all hits. Given the sheer size of our data set the registered buyer subsample still leads to 29.5 percent of the bilateral municipality flows to be non-zero (43,704 flows).

Table 1 shows that the sample size decreases when the actions are indicative of more serious housing search. The actions require different levels of effort from the users, while higher effort indicates more serious searchers. It seems that, at least at the aggregate level, these signals correctly reveal users as being more serious about their housing quest. As the opposite also holds, we will use the negating conditions (no account, no mortgage calculation, etc.) as a signal of recreational search.

## 4 Empirical model

We start by applying the gravity model described in Section 2 to our search flow context. Adjusting Eq (3) to search flows, i.e., the outflow of hits, and adding an error term leads to a fixed effects specification of the following form:

$$\begin{array}{c} ln\left(f_{ij}\right)=\beta_{0}+\beta_{1}I_{i}+\beta_{2}I_{j}+\beta_{3}ln\left(D_{ij}\right)+\beta_{4}M_{ij}+\beta_{5}C_{ij}+\beta_{6}R_{ij}+\epsilon_{it} \end{array}$$ (4)

where *f*_*ij*_ is the flow of hits from municipality *i* to municipality *j*, *I*_*i*_ and *I*_*j*_ are the origin and destination fixed effects (which include the multilateral resistance terms), *D*_*ij*_ is the distance between the centroids of municipality *i* and *j*, *M*_*ij*_ indicates whether the flow is internal (origin and destination municipality are the same), *C*_*ij*_ is contiguity (indicating whether municipalities are neighbors), *R*_*ij*_ indicates whether *i* and *j* are located in the same province (for *i* ≠ *j*), and *ϵ*_*it*_ is an error term. The common province variable captures regional and cultural similarities, including cultural identity and place attachment (for details on these topics, see [32, 33]). The origin fixed effects, *I*_*i*_, include the factors that encourage people to stay within their own municipality or to look elsewhere. The factors include, for instance, home bias in preferences [9] and the level of amenities. The destination fixed effects include pull factor related to, for instance, job opportunities and amenities at the destination municipality.

We have a cross section with two-dimensional fixed effects (i.e., origin and destination fixed effects). When the error term *ϵ*_*it*_ is idiosyncratic Eq (4) can easily be estimated with Least Squares Dummy Variables. However, Santos Silva and Tenreyro [34] demonstrate that heteroskedasticity is likely to be a problem when estimating gravity models, even when controlling for origin and destination fixed effects. More generally, they show that heteroskedasticity leads to biased estimates when a log-linearized model is estimated by OLS. They propose to estimate the non-log-linearized version of Eq (4); that is, they fall back to the multiplicative gravity equation as specified in Eq (1). They propose to estimate the model non-linearly with the Poisson pseudo maximum likelihood (PPML) estimator. An additional advantage of the PPML estimator is that it deals with the existence of zeros, whereas the logarithm of zero is undefined. In contrast to PPML, OLS estimation will thus lead to selection bias. In order to overcome the selection bias inherent to dropping zero observations, some authors suggest to add one (or another constant) to the dependent variable and using tobit for estimation. However, this leads to unit dependence and inconsistent estimates [12, 34].

Santos Silva and Tenreyro [34] show that the PPML estimator is numerically equal to their derived estimator; they do not argue to rely on count data estimators per se. Note, for instance, that negative-binomial and zero-inflated models are not scale-invariant. The only assumption that is needed for the PPML estimator to be consistent is that the conditional mean is correctly specified. The data do not have to be Poisson distributed nor do the data have to consist of integers [34, p. 645]. Santos Silva and Tenreyro [35] provide further simulation evidence and show that the PPML estimator is also ‘well behaved’ with many zeros in the sample; the proportions of zeros range from 0.62 to 0.83 in the simulations, which indicates that even our most restrictive sample, i.e., the registered buyers, can be used for estimation. In addition, Arvis and Shepherd [36] show another relevant property of the PPML estimator: the sum of the estimated trade flows are identical to the sum of the actual trade flows. In other words, PPML deals with the so-called ‘adding up’ problem. Arvis and Shepherd [36, p. 519] see this as another argument to use PPML as the “workhorse gravity model estimator”.

Fally [37] corroborates that the PPML estimator provides origin and destination fixed effects that are consistent with the multilateral resistance terms of Anderson and Van Wincoop [9]. Still, the PPML estimator for gravity equations is not entirely undisputed. Head and Mayer [12], for instance, suggest not to rely on PPML as the single estimator of gravity equations but to rely on multiple estimators. Stronger criticism can be found in, for instance, Burger et al. [38] and De Benedictis and Taglioni [39]. However, their criticism seems to be based on the misconception that over-dispersion and (larger than predicted) zero flows lead to inconsistent PPML estimates for gravity models [12]. Apart from that, the suggested alternatives—most notably, the negative binomial model—are receiving harsh criticism throughout. It is not without reason that Head and Mayer [12] “urge researchers to resist the siren song of the Negative Binomial” (p. 174) when used for the estimation of gravity models.

We use PPML as our preferred estimator. We also present OLS estimates of the log-linearized model with and without origin and destination fixed effects. The naive OLS specification, excluding the fixed effects, is informative because it includes origin and destination mass effects. The OLS specification with fixed effects provides insights into the bias of log-linearized estimation. With respect to the OLS estimates it is good to realise that selection bias is only a limited problem as zero flows are rare in most of our (sub)samples. Nevertheless, heteroskedasticity leads to inconsistent estimates for the log-linearized model even when the fixed effects are included.

## 5 Results

### 5.1 Main results

Table 2 provides the estimation results of Eq (4), and its multiplicative counterpart, for all platform users. Column 1 shows the results of the naive gravity model, estimated with OLS, which includes mass variables for both the origin and the destination. The mass of the origin is given by the natural log of the number of users in the origin municipality. The mass of the destination is given by the natural log of the number of objects in the destination. Using alternative mass variables for the origin and the destination in the naive specification, among which the total number of hits, the number of inhabitants, and the number of households, does not affect the findings. All else equal, the results indicate that a 1 percent increase in the number of users in the origin leads to a 1.1 percent increase in the outflow of hits, a 1 percent increase in the number of objects in the destination increases the flow to the municipality with 0.9 percent, and a 1 percent increase in distance decreases the flow with 0.7 percent. All else equal, flows within municipalities are 1,999 percent higher (100*[*exp*(3.044) − 1]) than external flows. Conversely, external flows are 95.2 percent lower (100*[*exp*(−3.044) − 1]) than internal flows. Wooldridge [40] stresses that the logarithmic approximation (i.e., multiplying the coefficients of the log-linear specification by 100) has the advantage that you do not have to specify the base group as the approximation will always fall within the range of the alternate base group percentages. Wooldridge thus suggests to state that the difference between internal and external flows is about 304 percent; internal flows are 1,999 percent higher than external flows, while external flows are 95.2 percent lower than internal flows. Sharing a municipality border leads to a flow that increases by 358 percent (100*[*exp*(1.521) − 1]) compared to when no border is shared. Being located in the same province increases flows by 59.7 percent (100*[*exp*(0.468) − 1]), ceteris paribus.

**Table 2:** Gravity model estimates of hit flows (full sample).

	(1)	(2)	(3)
	OLS	OLS (FE)	PPML (FE)
Log users origin	1.082***		
	(0.00240)		
Log objects destination	0.876***		
	(0.00333)		
Log distance	-0.736***	-0.983***	-1.127***
	(0.00506)	(0.00512)	(0.0276)
Within municipality	3.044***	2.220***	1.016***
	(0.0393)	(0.0406)	(0.131)
Contiguity	1.521***	1.055***	0.534***
	(0.0293)	(0.0273)	(0.0475)
Within province	0.468***	0.379***	0.121**
	(0.0112)	(0.00834)	(0.0467)
Constant	-7.099***	6.290***	12.64***
	(0.0444)	(0.0769)	(0.234)
Origin fixed effects	No	Yes	Yes
Destination fixed effects	No	Yes	Yes
Observations	147,903	147,903	148,216
R-squared	0.772	0.876	0.951

*Notes*: Standard errors clustered by municipality pair in parentheses.

* *p*<0.05,

** *p*<0.01,

*** *p*<0.001.

R-squared of the PPML estimation is calculated as the square of the correlation between observed and predicted hit flows.

Column 2 of Table 2 shows the OLS estimates where origin and destination fixed effects replace the mass variables. It is not possible to include both the mass variables and the fixed effects as the mass variables are constant within municipalities in our sample. After including fixed effects, the distance effect has become larger in magnitude: a 1 percent increase in distance reduces flows with 1.0 percent (ceteris paribus). The municipality, contiguity, and province coefficients have become smaller: within municipality flows increase by 821 percent, being neighbors increases flows by 187 percent, and being in the same province increases flows by 46.1 percent, all else equal.

Column 3 of Table 2 shows the results of the structural gravity model with our preferred estimator, i.e., the PPML estimates including origin and destination fixed effects. The PPML estimation is based on the full set of 148,216 flows as zero flows are also included. The absolute distance effect is shown to be even larger than in the previous estimations: a 1 percent increase in distance leads to a decrease in the flow of 1.1 percent, all else equal. The border coefficients (municipality, contiguity, and province) are substantially smaller but remain significant at the one percent level. Compared to their reference categories, within municipality flows lead to an increase of 176 percent, contiguity increases flows by 70.6 percent, and intra-provincial search leads to an increase of 12.9 percent.

In Table 3 the OLS fixed effects estimation results are shown for the groups that signal being potential house buyers. The findings are qualitatively the same for searchers with an account, searchers doing mortgage calculations, searchers checking the real estate agent’s telephone number, searchers receiving new listings by email, searchers sending messages to the real estate agent, searchers scheduling listings online, and those who registered as the buyer of a property. The number of observations (i.e., flows) decreases from 143, 997 for the users with an account to 122, 913 for users scheduling listings. The number of flows is significantly lower for the registered buyers: 43, 704. Similarly, the R-squared decreases from 0.789 for users with an account to 0.651 for users scheduling listings. It then falls to 0.409 for the registered buyers.

**Table 3:** Ordinary least squares estimates for serious searchers.

	(1)	(2)	(3)	(4)	(5)	(6)	(7)
	Account	Mortgage	Telephone	Email service	Message	Viewing	Buyer
	(22.3%)	(19.5%)	(21.0%)	(10.8%)	(8.0%)	(8.4%)	(0.4%)
Log distance	-1.058***	-1.076***	-1.120***	-1.070***	-1.086***	-1.170***	-0.613***
	(0.00594)	(0.00662)	(0.00636)	(0.00713)	(0.00772)	(0.00820)	(0.0142)
Within municipality	1.618***	2.066***	1.428***	1.814***	1.405***	1.428***	2.383***
	(0.0427)	(0.0471)	(0.0442)	(0.0491)	(0.0515)	(0.0554)	(0.0863)
Contiguity	0.846***	1.064***	0.798***	0.939***	0.857***	0.904***	1.013***
	(0.0259)	(0.0289)	(0.0266)	(0.0281)	(0.0282)	(0.0296)	(0.0447)
Within province	0.384***	0.412***	0.385***	0.424***	0.408***	0.425***	0.261***
	(0.0101)	(0.0111)	(0.0107)	(0.0120)	(0.0130)	(0.0137)	(0.0240)
Constant	4.826***	3.643***	4.630***	4.143***	2.986***	3.900***	1.744*
	(0.130)	(0.143)	(0.159)	(0.177)	(0.249)	(0.311)	(0.728)
Origin fixed effects	Yes	Yes	Yes	Yes	Yes	Yes	Yes
Destination fixed effects	Yes	Yes	Yes	Yes	Yes	Yes	Yes
Observations	143,997	142,317	142,072	135,050	126,757	122,913	43,704
R-squared	0.789	0.758	0.756	0.709	0.666	0.651	0.409

*Notes*: Standard errors clustered by municipality pair in parentheses.

* *p*<0.05,

** *p*<0.01,

*** *p*<0.001.

R-squared of the PPML estimation is calculated as the square of the correlation between observed and predicted hit flows. The column name indicates the subsample of users that is conditioned on. Account, users that have registered and created a user account; Mortgage, users that have done the online mortgage calculation; Telephone, users that have pressed the button for the real estate agent’s telephone number; Email service, users that have signed up for an email service of new listings within their preferences; Message, users that have contacted the real estate agent through an online form; Viewing, users that scheduled a viewing with the online tool; Buyer, users that registered themselves as the buyer of a property.

The estimates for the first six subgroups in Table 3 show mostly moderate differences. Insofar differences do exist, the coefficients do not indicate a clear pattern related to increasing (or decreasing) seriousness among these users. The distance coefficients in the columns 1 to 6 are between -1.170 and -1.058, indicating that a 1 percent increase in distance decreases hit flows by between 1.1 and 1.2 percent, ceteris paribus. The coefficient indicating internal flows ranges between 1.405 and 2.066, indicating increases of between 308 and 689 percent. The contiguity coefficients are between 0.798 and 1.064, indicating that being a neighbor increases flows by between 122 and 190 percent, ceteris paribus. The province coefficients are between 0.384 and 0.425; that is, all else equal, being located in the same province increases hit flows by between 46.8 and 53.0 percent.

Column 7 of Table 3 shows that the estimates for the registered buyers are quantitatively different. The coefficients for the distance effect (-0.613) and the province effect (0.261) indicate substantially smaller magnitudes. In contrast, the municipality effect is larger (2.383). The contiguity coefficient does fall within the boundaries of the other groups. The signs of the coefficients for registered buyers are thus the same as for the other groups while they remain highly significant.

Table 4 shows the estimation results of the users that have not signaled being serious searchers. For practical reasons, we refer to these non-serious searchers as the recreational searchers. As the estimates are based on larger numbers of hits, the results are closer to the full-sample estimates. They also show less variation between the groups. Particularly, the results excluding the registered buyers are virtually the same as those of the full sample as it involves 99.6 percent of the hits of the full sample. The distance coefficient for the recreational searchers ranges between -0.969 and -0.983 suggesting that the absolute effect is smaller than that of the serious searchers. Overall, recreational searchers are likelier to search within their municipality (coefficients between 2.221 and 2.375) and in contiguous municipalities (coefficients between 1.054 and 1.116) than serious searchers. Still, it seems that they search less intra-provincially (coefficients between 0.379 and 0.388).

**Table 4:** Ordinary least squares estimates for recreational searchers.

	(1)	(2)	(3)	(4)	(5)	(6)	(7)
	No account	No mortgage	No telephone	No email	No message	No viewing	Non-buyer
	(77.7%)	(80.5%)	(79.0%)	(89.2%)	(92.0%)	(91.6%)	(99.6%)
Log distance	-0.982***	-0.979***	-0.969***	-0.981***	-0.979***	-0.974***	-0.983***
	(0.00530)	(0.00514)	(0.00526)	(0.00518)	(0.00517)	(0.00513)	(0.00512)
Within municipality	2.342***	2.249***	2.375***	2.261***	2.276***	2.276***	2.221***
	(0.0417)	(0.0407)	(0.0414)	(0.0411)	(0.0408)	(0.0408)	(0.0406)
Contiguity	1.106***	1.054***	1.116***	1.074***	1.078***	1.073***	1.055***
	(0.0283)	(0.0275)	(0.0282)	(0.0276)	(0.0276)	(0.0275)	(0.0273)
Within province	0.387***	0.381***	0.388***	0.379***	0.380***	0.379***	0.379***
	(0.00856)	(0.00837)	(0.00851)	(0.00843)	(0.00841)	(0.00836)	(0.00834)
Constant	5.850***	4.562***	7.986	5.907***	4.662***	4.638***	5.514***
	(0.0851)	(0.0906)	(34.07)	(0.0847)	(0.0923)	(0.0925)	(0.0823)
Origin fixed effects	Yes	Yes	Yes	Yes	Yes	Yes	Yes
Destination fixed effects	Yes	Yes	Yes	Yes	Yes	Yes	Yes
Observations	147,796	147,820	147,815	147,864	147,880	147,884	147,902
R-squared	0.868	0.874	0.872	0.873	0.874	0.875	0.876

*Notes*: Standard errors clustered by municipality pair in parentheses.

* *p*<0.05,

** *p*<0.01,

*** *p*<0.001.

R-squared of the PPML estimation is calculated as the square of the correlation between observed and predicted hit flows. The column name indicates the subsample of users that is conditioned on. Account, users that have registered and created a user account; Mortgage, users that have done the online mortgage calculation; Telephone, users that have pressed the button for the real estate agent’s telephone number; Email service, users that have signed up for an email service of new listings within their preferences; Message, users that have contacted the real estate agent through an online form; Viewing, users that scheduled a viewing with the online tool; Buyer, users that registered themselves as the buyer of a property.

Table 5 shows the PPML estimation results of the structural gravity model for the serious searchers. It is important to note that zero flows are included as it explains why the results for the registered buyers do no longer stand out. Comparing the registered buyers sample with the other samples shows that the coefficients for distance, municipality, and province fall within the range of the other groups. Only the province coefficient is larger. The PPML estimates for the distance effect are similar to the earlier OLS estimates. The distance coefficient ranges between -1.190 and -1.101 for serious searchers, suggesting that, all else equal, a 1 percent increase in distance leads to a decrease in the flow of between 1.1 and 1.2 percent. In contrast, the estimated municipality, contiguity and province effects are substantially smaller in the PPML estimation. The municipality coefficient is between 0.490 and 0.982, suggesting an increase of between 63.2 and 167 percent when flows are within a municipality. The contiguity coefficient is between 0.378 and 0.543, leading to an increase of 45.9 to 72.1 percent for contiguous flows, all else equal. The province coefficient ranges from 0.0973 to 0.195, implying increases of 10.2 and 21.5 percent, respectively, when flows are within the same province. The differences between the OLS estimates of Table 3 and the PPML estimates of Table 5 demonstrate the importance of estimating the model multiplicatively.

**Table 5:** Poisson pseudo maximum likelihood estimates for serious searchers.

	(1)	(2)	(3)	(4)	(5)	(6)	(7)
	Account	Mortgage	Telephone	Email service	Message	Viewing	Buyer
	(22.3%)	(19.5%)	(21.0%)	(10.8%)	(8.0%)	(8.4%)	(0.4%)
Log distance	-1.101***	-1.146***	-1.113***	-1.134***	-1.117***	-1.190***	-1.141***
	(0.0275)	(0.0281)	(0.0282)	(0.0338)	(0.0301)	(0.0305)	(0.0311)
Within municipality	0.754***	0.982***	0.668***	0.698***	0.490***	0.513***	0.880***
	(0.132)	(0.127)	(0.133)	(0.159)	(0.140)	(0.135)	(0.124)
Contiguity	0.433***	0.543***	0.424***	0.413***	0.378***	0.391***	0.440***
	(0.0528)	(0.0503)	(0.0552)	(0.0597)	(0.0625)	(0.0612)	(0.0661)
Within province	0.145**	0.145**	0.138**	0.0973	0.103*	0.131**	0.195***
	(0.0469)	(0.0478)	(0.0474)	(0.0593)	(0.0512)	(0.0502)	(0.0515)
Constant	10.91***	11.11***	10.72***	9.798***	9.491***	9.939***	6.016***
	(0.209)	(0.230)	(0.208)	(0.260)	(0.243)	(0.291)	(0.490)
Origin fixed effects	Yes	Yes	Yes	Yes	Yes	Yes	Yes
Destination fixed effects	Yes	Yes	Yes	Yes	Yes	Yes	Yes
Observations	148,216	148,216	148,216	148,216	148,216	148,216	148,216
R-squared	0.974	0.954	0.967	0.975	0.981	0.976	0.934

*Notes*: Standard errors clustered by municipality pair in parentheses.

* *p*<0.05,

** *p*<0.01,

*** *p*<0.001.

R-squared of the PPML estimation is calculated as the square of the correlation between observed and predicted hit flows. The column name indicates the subsample of users that is conditioned on. Account, users that have registered and created a user account; Mortgage, users that have done the online mortgage calculation; Telephone, users that have pressed the button for the real estate agent’s telephone number; Email service, users that have signed up for an email service of new listings within their preferences; Message, users that have contacted the real estate agent through an online form; Viewing, users that scheduled a viewing with the online tool; Buyer, users that registered themselves as the buyer of a property.

Table 6 shows the PPML estimation results for recreational searchers. The estimated distance coefficient is between -1.130 and -1.119. For three of the subgroups the distance coefficient is larger than for the opposite—i.e., serious—subgroup and for four of the subgroups it is smaller than for the opposite subgroup. One has to conclude that the distance effects of serious and recreational searchers are almost identical. Nevertheless, the municipality coefficients do differ. With an estimated coefficient of between 1.017 and 1.113 (176 and 204 percent, respectively), the effect is larger for recreational searchers in all subsamples. The coefficient of contiguity is between 0.532 and 0.568 (70.2 and 76.5 percent, respectively), which implies that recreational platform users search more often in neighboring municipalities—the mortgage versus no mortgage calculation being the exception. Furthermore, the estimates seem to indicate that recreational users search relatively less intra-provincially. The coefficient is between 0.116 and 0.125 (between 12.3 and 13.3 percent) and it is smaller than the point estimates for serious searchers in five out of the seven estimations.

**Table 6:** Poisson pseudo maximum likelihood estimates for recreational searchers.

	(1)	(2)	(3)	(4)	(5)	(6)	(7)
	No account	No mortgage	No telephone	No email	No message	No viewing	Non-buyer
	(77.7%)	(80.5%)	(79.0%)	(89.2%)	(92.0%)	(91.6%)	(99.6%)
Log distance	-1.130***	-1.123***	-1.126***	-1.124***	-1.125***	-1.119***	-1.127***
	(0.0278)	(0.0277)	(0.0277)	(0.0270)	(0.0274)	(0.0275)	(0.0277)
Within municipality	1.098***	1.024***	1.113***	1.056***	1.067***	1.065***	1.017***
	(0.130)	(0.133)	(0.130)	(0.128)	(0.130)	(0.131)	(0.131)
Contiguity	0.568***	0.532***	0.568***	0.550***	0.551***	0.549***	0.535***
	(0.0470)	(0.0471)	(0.0471)	(0.0465)	(0.0470)	(0.0470)	(0.0475)
Within province	0.118*	0.116*	0.120**	0.125**	0.125**	0.122**	0.121**
	(0.0464)	(0.0465)	(0.0462)	(0.0451)	(0.0461)	(0.0463)	(0.0468)
Constant	12.43***	12.40***	12.46***	12.57***	12.58***	12.55***	12.64***
	(0.243)	(0.237)	(0.242)	(0.233)	(0.235)	(0.232)	(0.234)
Origin fixed effects	Yes	Yes	Yes	Yes	Yes	Yes	Yes
Destination fixed effects	Yes	Yes	Yes	Yes	Yes	Yes	Yes
Observations	148,216	148,216	148,216	148,216	148,216	148,216	148,216
R-squared	0.939	0.950	0.944	0.944	0.945	0.946	0.951

*Notes*: Standard errors clustered by municipality pair in parentheses.

* *p*<0.05,

** *p*<0.01,

*** *p*<0.001.

R-squared of the PPML estimation is calculated as the square of the correlation between observed and predicted hit flows. The column name indicates the subsample of users that is conditioned on. Account, users that have registered and created a user account; Mortgage, users that have done the online mortgage calculation; Telephone, users that have pressed the button for the real estate agent’s telephone number; Email service, users that have signed up for an email service of new listings within their preferences; Message, users that have contacted the real estate agent through an online form; Viewing, users that scheduled a viewing with the online tool; Buyer, users that registered themselves as the buyer of a property.

### 5.2 Robustness

We have noted before that the geolocation that is used in our analyses can differ from the true physical user location because of two reasons. First, geolocations are approximations of the true location. Second, the geolocations that we use are based on anonymized IP-addresses. Aggregating smaller locations into municipalities, as we have done, does not eliminate the measurement error fully. Therefore, we will test the robustness of the results by looking into the effect of measurement error in the user location. After all, errors in the user location lead to measurement error in our flow data. In this section, we will estimate additional gravity models that are based on different samples. The subsamples, based on the user’s device and network domain, allow us to test whether the aforementioned errors in the user location can drive our results.

Table 7 divides the underlying observations into three subsamples based on the device that is used by the platform user. We do so because location precision, the difference between the true physical location and the assigned geolocation, is device specific. Mobile devices are said to have higher precision because of triangulation between cell towers and (indirect) usage of GPS [41]. The device categories are desktop (including laptop users), tablet, and mobile. The categories of most interest are desktop and mobile as the difference in the network type that is used, computer network versus cellular network, is largest between these categories. Furthermore, the technical features between these types of devices differ most. We consider the tablets as the intermediately category as, for instance, they may or may not have a GPS chip.

**Table 7:** Estimation results for device categories.

	OLS (FE)			PPML (FE)		
	Desktop	Tablet	Mobile	Desktop	Tablet	Mobile
	(1)	(2)	(3)	(4)	(5)	(6)
Log distance	-0.969***	-1.044***	-1.056***	-1.067***	-1.133***	-1.170***
	(0.00514)	(0.00618)	(0.00603)	(0.0244)	(0.0212)	(0.0355)
Within municipality	1.970***	2.331***	2.431***	0.953***	1.398***	1.148***
	(0.0391)	(0.0465)	(0.0453)	(0.115)	(0.0892)	(0.158)
Contiguity	0.922***	1.138***	1.188***	0.458***	0.652***	0.647***
	(0.0255)	(0.0294)	(0.0308)	(0.0452)	(0.0401)	(0.0561)
Within province	0.380***	0.414***	0.410***	0.184***	0.245***	0.0521
	(0.00847)	(0.0103)	(0.00983)	(0.0392)	(0.0316)	(0.0592)
Constant	3.931***	10.80	4.924***	11.67***	11.15***	11.48***
	(0.101)	(258.8)	(0.103)	(0.196)	(0.247)	(0.288)
Origin fixed effects	Yes	Yes	Yes	Yes	Yes	Yes
Destination fixed effects	Yes	Yes	Yes	Yes	Yes	Yes
Observations	147,147	144,971	146,324	148,216	148,216	148,216
R-squared	0.853	0.791	0.829	0.979	0.937	0.922

*Notes*: Standard errors clustered by municipality pair in parentheses.

* *p*<0.05,

** *p*<0.01,

*** *p*<0.001.

R-squared of the PPML estimation is calculated as the square of the correlation between observed and predicted hit flows.

In Table 7 we focus on the distance coefficients as those are of main interest. Columns 1 and 3 give the OLS estimates when origin and destination fixed effects are included. The distance coefficient for desktop devices is -0.969 compared to -1.056 for mobile devices, while the coefficient for tablets falls between the two. The results thus suggest that measurement error leads to attenuation bias (bias towards zero); the higher location precision for mobile devices leads to a slightly larger absolute effect for distance. The PPML estimates in columns 4 and 6 suggest the same. The distance coefficient is -1.067 for desktop devices and -1.170 for mobile devices. Again, attenuation bias seems to reduce the coefficient for desktop devices. Hence, the results in Table 7 suggest that the estimates of the distance effect that we have presented thus far are in fact lower bounds. The actual distance effects are expected to be larger as our estimates are affected by attenuation bias.

Table 8 splits the sample based upon the network domain that is used. We divide the network domain into two categories based on whether or not the label (i.e., value) is given as *not set*. (Strictly speaking, the category contains parentheses in Google Analytics: *(not set)*.) We create the category not set and the category not not set, which we will refer to as set. This division allows us to investigate the extent to which the IP anonymization, the dropping of the last three digits of the full IP address, affects the estimates. The effect of anonymization differs between network domains (see subsection 3.2). Most importantly, IP anonymization does virtually not affect the geolocation of hits where the network domain is not set (see Appendix).

**Table 8:** Estimation results for network domain.

	OLS (FE)		PPML (FE)	
	Domain set	Domain not set	Domain set	Domain not set
	(1)	(2)	(3)	(4)
Log distance	-0.940***	-1.398***	-1.086***	-1.475***
	(0.00518)	(0.00714)	(0.0281)	(0.0291)
Within municipality	2.246***	1.633***	0.968***	0.977***
	(0.0423)	(0.0603)	(0.139)	(0.102)
Contiguity	1.031***	0.912***	0.495***	0.560***
	(0.0275)	(0.0328)	(0.0475)	(0.0575)
Within province	0.366***	0.496***	0.0781	0.372***
	(0.00840)	(0.0121)	(0.0478)	(0.0448)
Constant	5.977***	5.660***	12.43***	10.92***
	(0.0783)	(0.275)	(0.236)	(0.417)
Origin fixed effects	Yes	Yes	Yes	Yes
Destination fixed effects	Yes	Yes	Yes	Yes
Observations	147,872	127,763	148,216	148,216
R-squared	0.867	0.784	0.943	0.978

*Notes*: Standard errors clustered by municipality pair in parentheses.

* *p*<0.05,

** *p*<0.01,

*** *p*<0.001.

R-squared of the PPML estimation is calculated as the square of the correlation between observed and predicted hit flows.

The estimates in Table 8 suggest that IP anonymization also leads to attenuation bias. The estimated absolute distance effect is smaller for the set category than for the not set category. This holds for both the OLS estimates (-0.940 versus -1.398) and the PPML estimates (-1.086 versus -1.475). Again, this suggests that the true distance effect is larger than what we have estimated before. Nevertheless, we suggest to interpret these results with some caution as the user domain is likely to be correlated with both observables and unobservables of the users. In terms of the previous device categories: desktop users, tablet users, and mobile users are likely to differ in more aspects than only in the device they use.

## 6 Conclusions

In this paper we have applied a gravity framework to user-generated data of Funda, the largest housing market platform in the Netherlands. In the full sample we have information on 148,216 flows, among which 313 zero flows, from one municipality to another. These flows are based on an underlying data set of 1.097 billion hits on 279,941 objects (houses and apartments) in 388 municipalities. We show that gravity describes the patterns of inflow and outflow of hits from one municipality to another, where the location of the user defines the origin and the location of the viewed property defines the destination. We demonstrate that even a naive gravity model explains close to 80 percent of all flows. Evidently, the explanatory power of the model rises further when origin and destination fixed effects are added.

Estimation of the gravity model through PPML, based on the full sample of hits that includes serious searchers, recreational searchers, and everything in between, indicates that a 1 percent increase in the distance between municipalities decreases the search flow with 1.1 percent, ceteris paribus. The municipality, contiguity, and province coefficients indicate that border effects matter. Search within the municipality increases the size a the flow by 176 percent, being neighboring municipalities leads to an increase of 71 percent, and being located in the same province increases the size of the search flow by 13 percent.

We distinguish serious searchers from recreational searchers by paying attention to platform users that signal being serious. We demonstrate that the gravity framework describes search patterns of both types of searchers. Looking at border effects, the results indicate that recreational search is centered more around the user’s location: recreational searchers have a relative preference for properties within the origin municipality and its neighbors while serious searchers have a relative preference for other municipalities within the province border. Still, most importantly, the results do not indicate differences between the distance effects of serious and recreational searchers.

The effect of measurement error in the geolocation was investigated by splitting the data set based on devices and network domains. Together they are informative about the effect of differences between the true locations and the geolocations based on anonymized IP addresses, which are used for privacy protection of the users in the data set. The results indicate that attenuation bias is present in our estimates; that is, the results suggest that our estimates can be considered a lower bound of the true absolute distance effect.

All in all, we find that distance is important in online housing search for both serious and recreational searchers. The distance effect does not differ between these groups even though differences in border effects exist. The findings demonstrate that the gravity framework is relevant for both serious and recreational searchers.

The similarities between serious and recreational searchers make even aggregated platform data relevant for policymakers. Housing market trends can be identified earlier as search precedes transactions. It can also provide policy makers and land planners with information on what locations (and property characteristics) are in highest demand. The most important societal benefit is that an improved understanding of online search can lower search costs and achieve better matches between buyers and properties. However, we do want to stress that policy applications should not be achieved at the cost of user privacy.

The international trade literature is increasingly including Internet trade and even digital goods. It is important that this development continues. In the housing search context, future research could explicitly incorporate spatial dependencies. Low-aggregation data, like ours, seems promising for this objective. Still most of all, we hope that future gravity research will focus more often on information flows. Without a doubt, information flows have the future.

## 7 Appendix: IP anonymization

Clifton and Wan [42] have assessed the impact of Google’s Anonymize IP (AIP) based on international data from 957,253 sessions that were collected in May and June 2018. For the Netherlands, they find that the anonymization tool does not affect the location, at the lowest geographical level, in 66.40 percent of the cases. It should be noted that this is referred to as “city” by Google Analytics even though the great majority are in fact villages. The assessment shows significant differences between “cities”. For instance, the assessment shows an accuracy of 86.9 percent for Amsterdam, 73.9 percent for The Hague, 69.1 percent for Utrecht, and 67.0 percent for Rotterdam.

We use the data from Clifton and Wan to look into the measurement error that arises when IP addresses are anonymized. We restrict the observations to those where the locations of both the anonymized and the full IP are found within the Netherlands. After all, Funda users are supposed to be located in the Netherlands. We thus exclude sessions that are (erroneously) provided with a foreign location as we do in our regression samples. We are left with a data set of 27,589 sessions. As our analysis relies on municipalities instead of villages and cities, we re-code them into municipalities, which increases the accuracy rates: the overall accuracy rate, at the municipality level, is 69.6 percent. We are missing data for the same six municipalities as in our main data set (Ameland, Schiermonnikoog, Vlieland, Rozendaal, Haarlemmerliede en Spaarnwoude, and De Marne).

**Fig 5:**
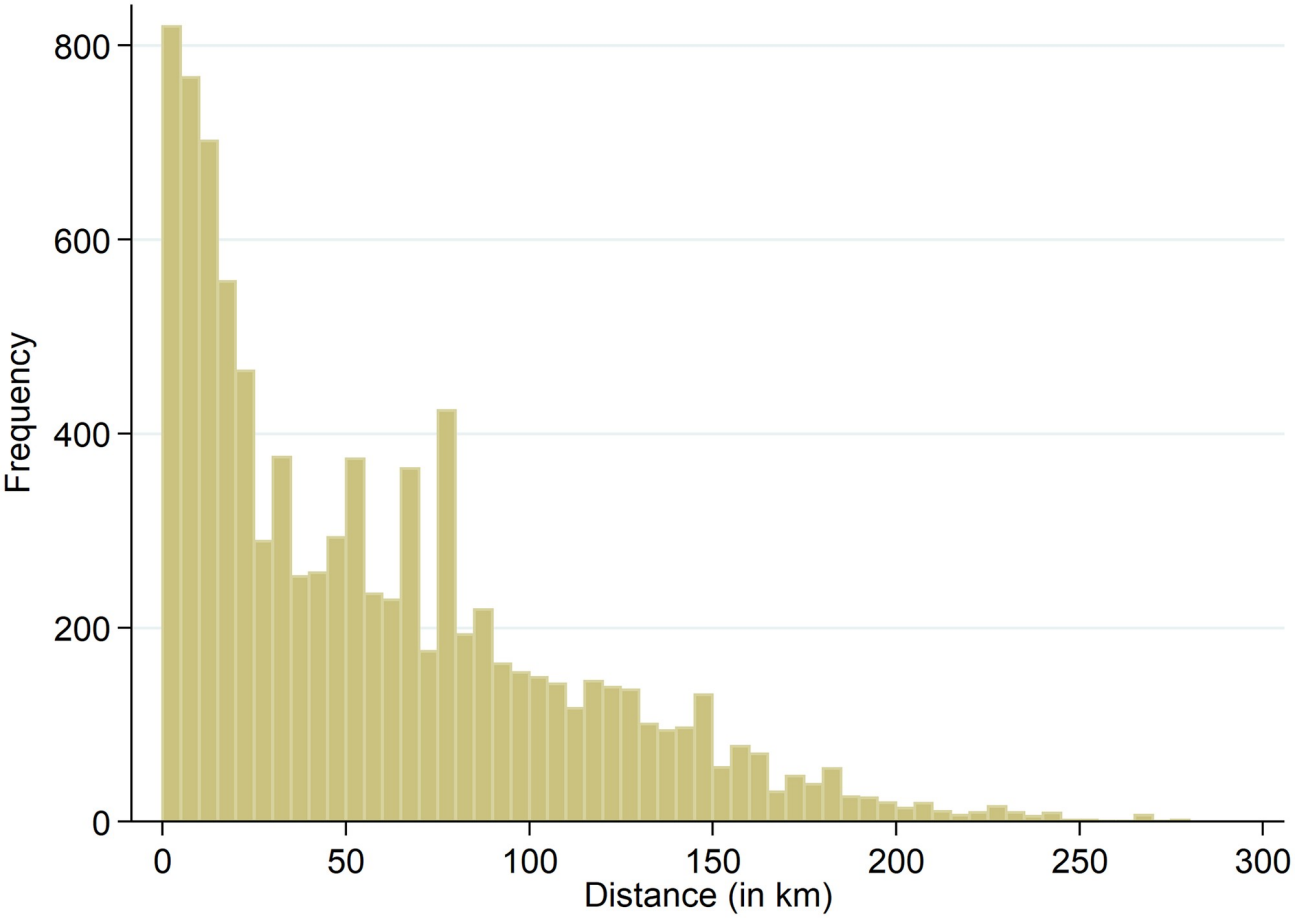
Non-zero distances between full-IP and anonymized geolocations at session level.

**Fig 6:**
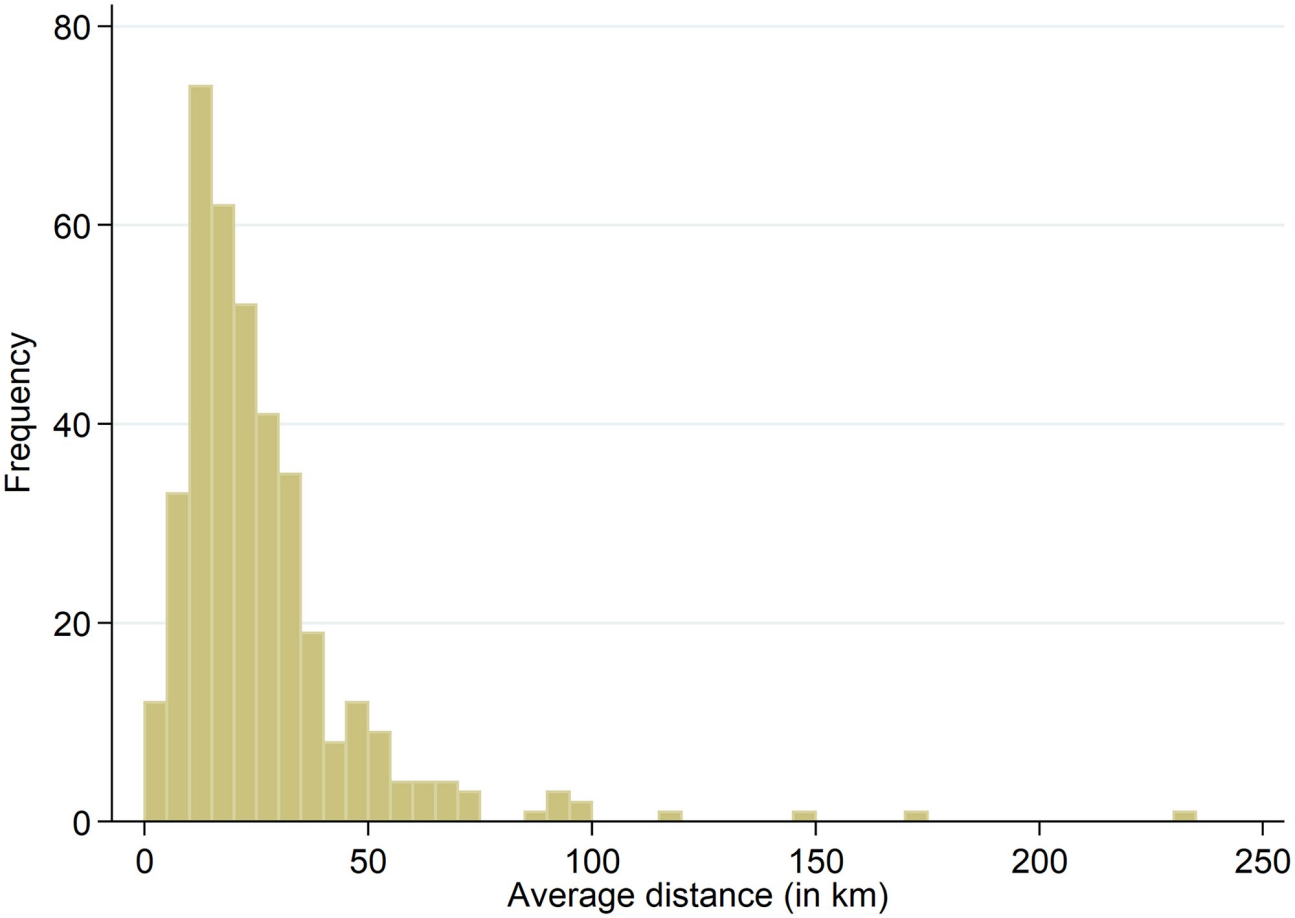
Average distance between full-IP and anonymized geolocations per municipality.

The average distance between the full-IP and anonymized geolocations for the total 27,589 observations is 19.18 km. The distribution of the non-zero distances, consisting of 30.4 percent of the data (8,374 observations), is shown in Fig 5. Note that for comparibility reasons we use a fixed bar width of 5.0 km in this and the following histograms. The figure shows that frequencies decrease for larger errors. The average distance between the geolocations for the 8,374 non-zero observations is 63.18 km (the median is 52.82 km). Averaging the errors at the municipality level leads to Fig 6. The figure shows that the largest group has an average error of 10-15 km. Four municipalities have an average error larger than 100 km; nevertheless, these averages are based on between 1-5 observations only.

**Fig 7:**
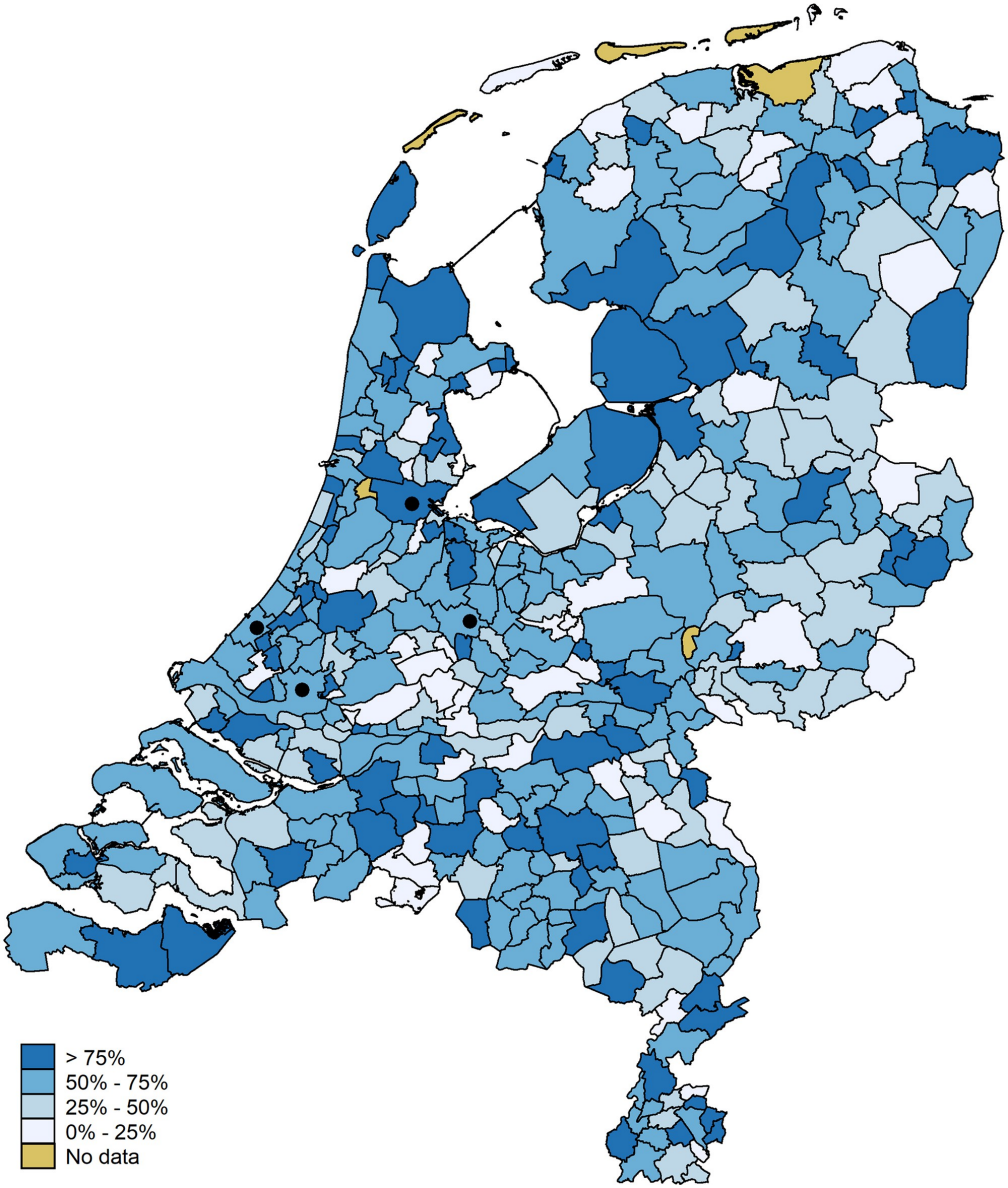
Location accuracy per municipality. Digital geometry (i.e., the shapefile) obtained from CBS/Kadaster [30]).

**Fig 8:**
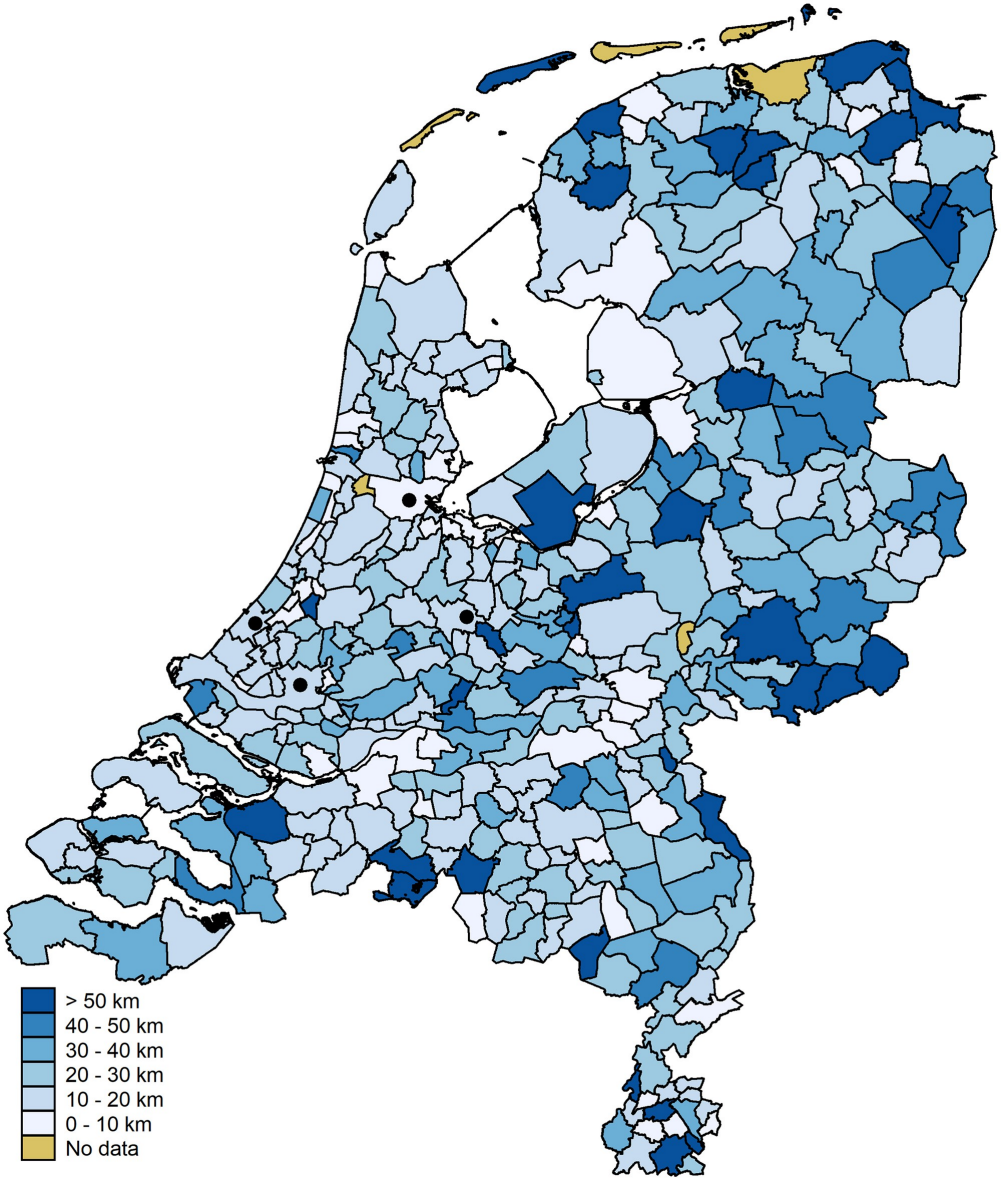
Average distance error per municipality. Digital geometry (i.e., the shapefile) obtained from CBS/Kadaster [30]).

As we focus on spatial relationships we also present the data mapwise. Fig 7 shows the accuracy per municipality once more. The municipalities of the four large cities are indicated with a black dot. The municipality of Amsterdam falls in the highest category (accuracy of 87.5 percent). The municipalities of The Hague, Utrecht, and Rotterdam fall in the second category (accuracies of 74.4, 71.9, and 68.9 percent, respectively). A substantial part of the variation between municipalities is driven by small numbers of observations; that is, municipalities with small observation numbers tend to have extreme values (five municipalities have an accuracy of 100 percent, 17 have an accuracy of zero). Fig 8 shows the information from Fig 6 on a map. We stress, once more, that the errors above 50 km are driven by small numbers of observations.

As noted before, the effect of the anonymization tool depends, among other things, on the Internet Service Provider (ISP). The ISP can, for example, be identified though the network domain, which is one of the available dimensions. The network domain thus provides information on the ISPs in the sample. These include Ziggo (21.3 percent), Direct-adsl (7.1 percent), and KPN.net (6.7 percent). The most interesting from our perspective is the category *not set* (14.6 percent). As is turns out, the location accuracy of this category is almost unaffected by the IP anonymization.

**Fig 9:**
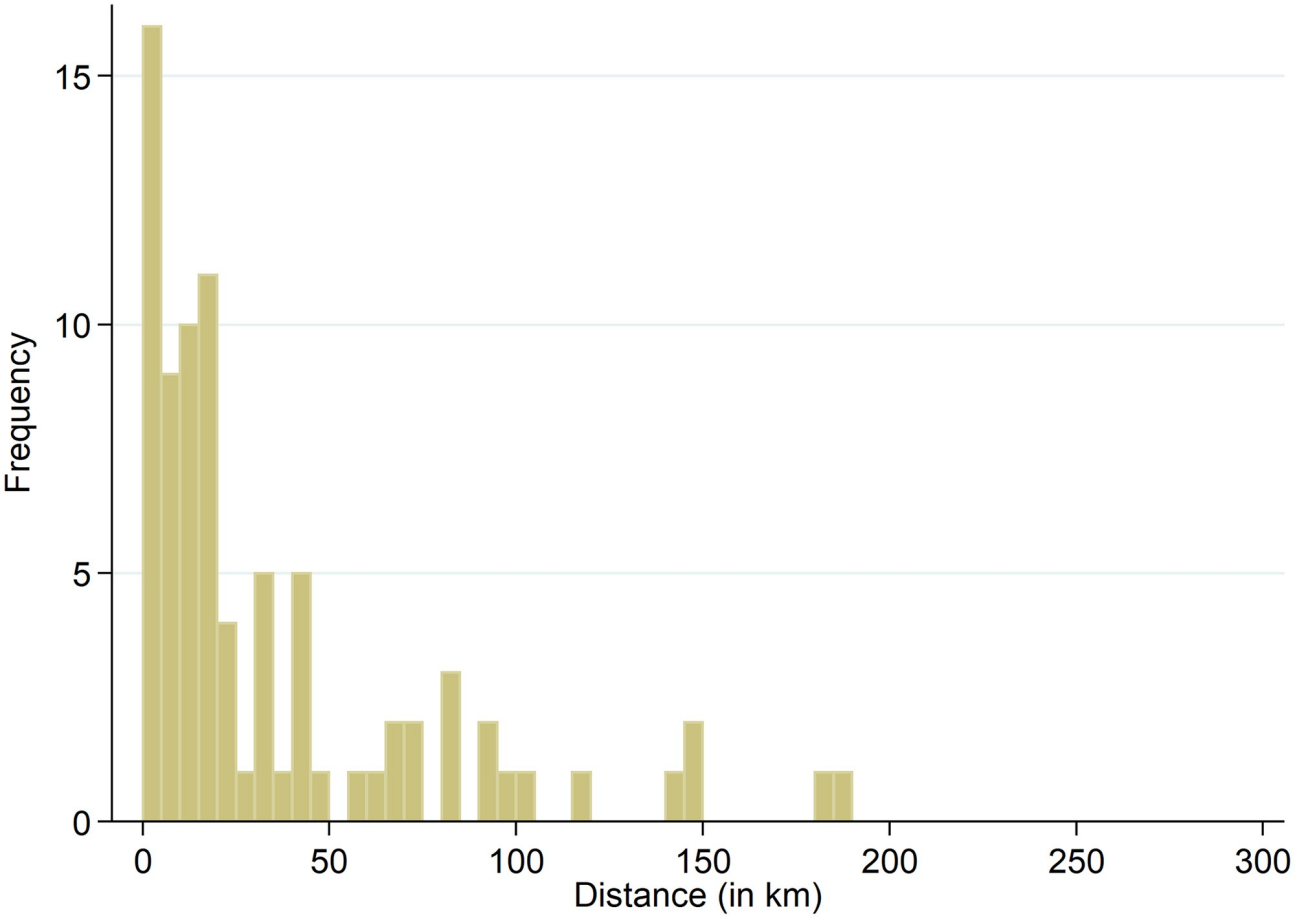
Non-zero distances at session level for network domain *not set*.

**Fig 10:**
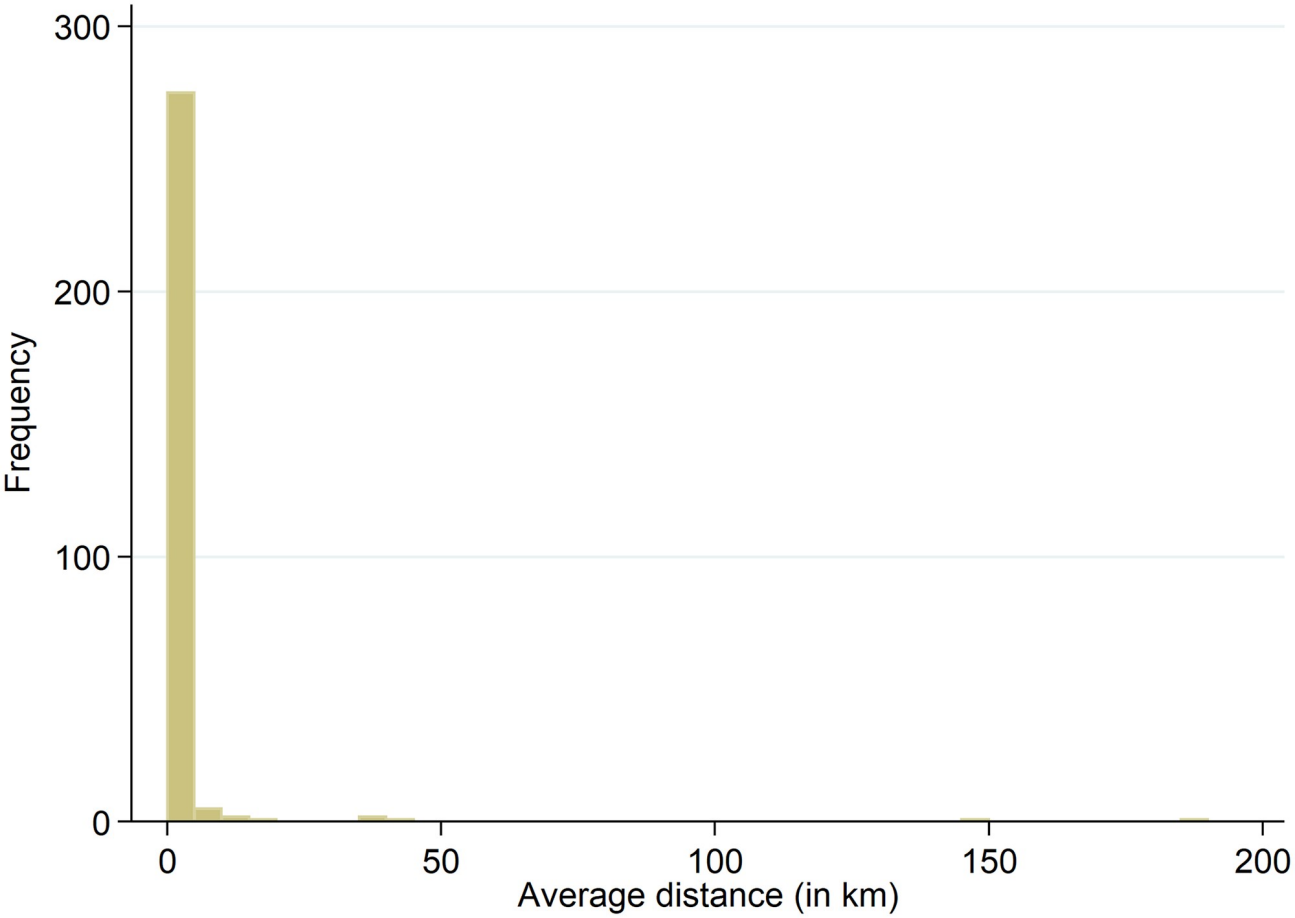
Average distance per municipality for network domain *not set*.

We have in total 4,022 observations where the network domain is *not set*. The average distance between the geolocations for this sample is only 0.72 km. This is mainly due to the high accuracy: for 98.4 percent of the observations the anonymization does not affect the geolocation. For 66 observations (1.6 percent) the municipality based on the full-IP and the anonymized IP does differ. The average distance error between the geolocations for the non-zero observations is 44.04 km (the median is 24.58 km). Fig 9 shows the distribution of the non-zero distances for these observations. Fig 10 shows the average error per municipality. The figure shows that 275 municipalities have an average error of at most 5 km; only 13 municipalites have an average error that is larger than 5.0 km.

**Fig 11:**
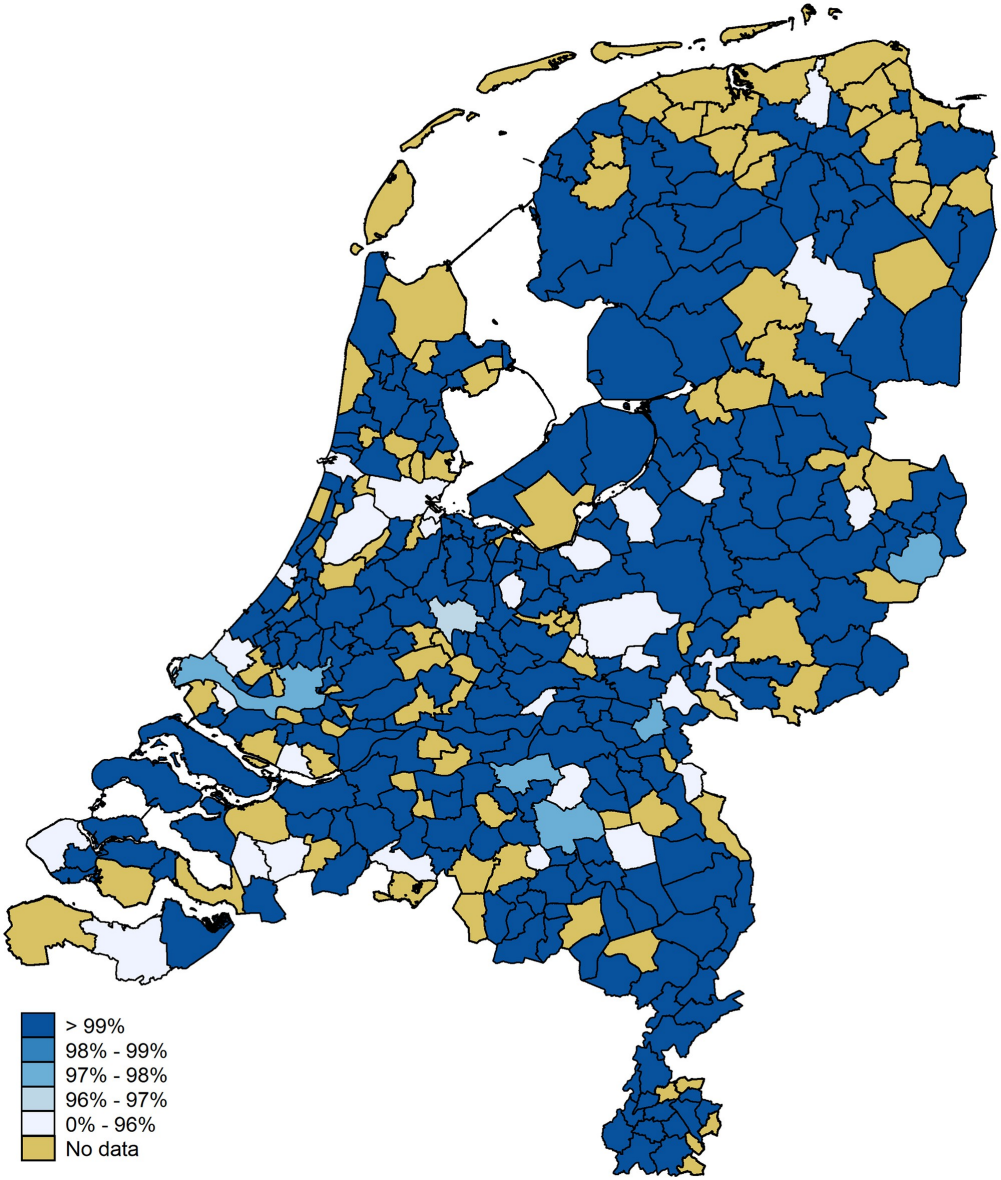
Location accuracy per municipality for network domain *not set*. Digital geometry (i.e., the shapefile) obtained from CBS/Kadaster [30]).

**Fig 12:**
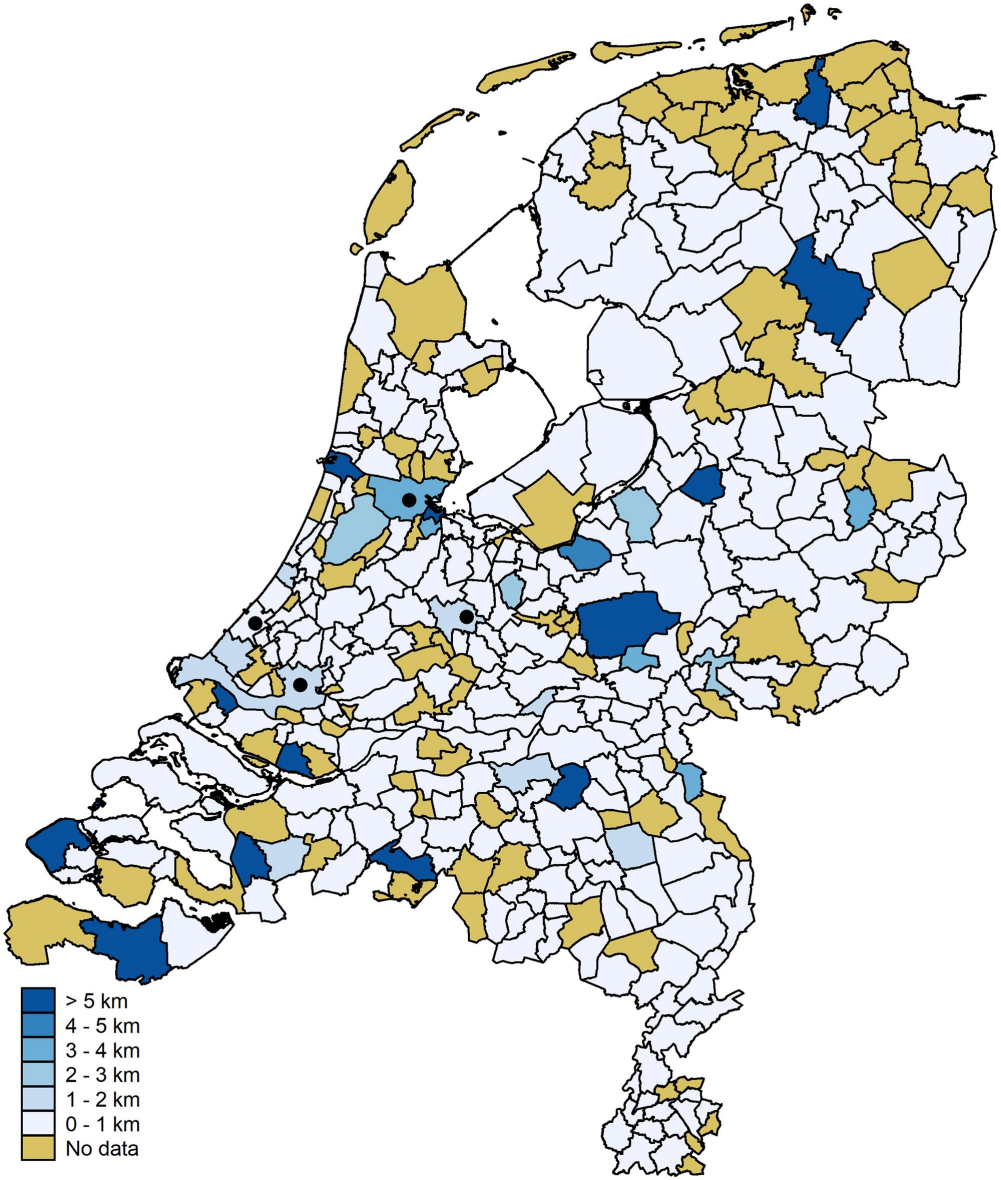
Average distance error per municipality for network domain *not set*. Digital geometry (i.e., the shapefile) obtained from CBS/Kadaster [30]).

The mapwise statistics are plotted in Figs 11 and 12. Fig 11 shows that, due to the smaller number of observations, no data are available for 94 municipalities (compared to six before). Out of the remaining 288 municipalities only 30 (10.4 percent) have an accuracy rate below 96 percent. Fig 12 shows the average error between the full-IP and anonymized IP geolocations. Most importantly, 13 municipalities have an average distance above 5.0 km. Still, these relatively large average errors are, again, driven by small numbers of observations: 7 of these municipalities have observation numbers between 1 and 4.

All in all, the observations where the network domain is *not set* show limited effects of the Google anonymization tool. The accuracy rates based on the anoymized IP are high and the average distances to the full-IP geolocations are small. It is for that reason that we use this particular subsample for robustness purposes.
